# Fungus: how to prevent growth and remove it from optical components

**Published:** 2013

**Authors:** Ismael Cordero

**Affiliations:** Clinical engineer: New York, NY, USA **ismaelcordero@me.com**

**Figure:**
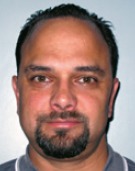
Ismael Cordero

In hot and humid climates it is common for fungus to grow on the surfaces of optical components. Airborne fungal spores settle on optical surfaces and develop into organisms that digest organic material, such as oils from fingerprints or lens coatings, producing hydrofluoric acid as a waste product. This acid in turn destroys any lens coatings and permanently etches the glass.

**Figure:**
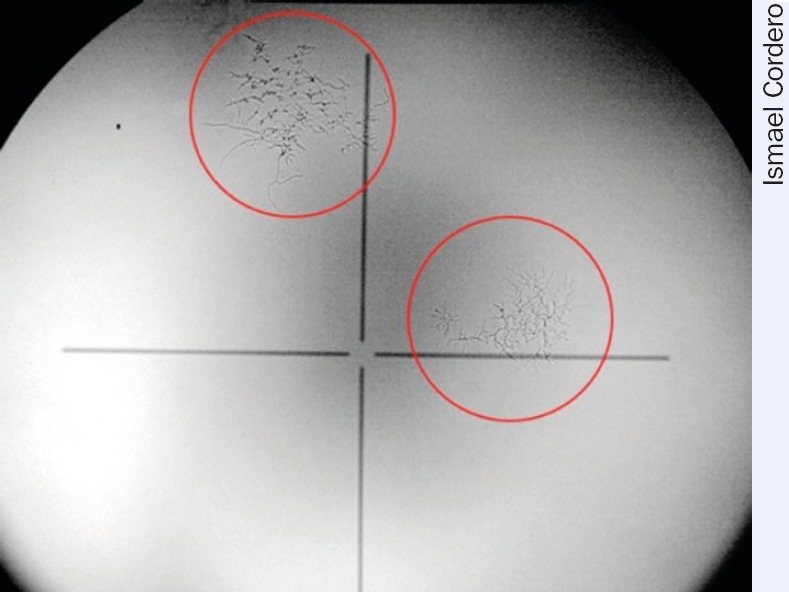
Figure 1

In its initial stages, fungus, as pictured in Figure 1, may not be perceivable by the clinician. However, over time the fungus will cover the lens surface in a web-like manner, initially causing a very slight loss of image brightness, followed by decreased contrast due to light reflecting off the fungus. In its final stages, the fungus etches the outer coatings of the lens and image sharpness deteriorates.

## Preventing fungal growth

Fungal spores are everywhere and germinate under suitable environmental conditions:

- relative humidity of at least 70% for more than 3 days
- little or no airflow
- darkness
- nutrients (textile lint, traces of grease, varnish, dust and dirt)

To prevent fungal growth on optical components the following precautions should be observed.

- After instruments with optics have been used and cleaned, they should be dried immediately. Turning on a fan in the room will hasten the drying process.
- Keep optical components in a dry place with a relative humidity of less than 65% and with plenty of air circulation. Air conditioners and dehumidifiers are very helpful, but must be used 24 hours a day since sudden changes in temperature and humidity promote the growth of fungus.
- In a humid environment, do not cover optics with plastic drape covers (commonly supplied by medical equipment manufacturers) since these will retain humidity. If you need to drape the equipment to protect against dust, use a cloth cover. Do not use containers made of leather, textiles or wood to store optics.
- You can keep optics in sealed plastic containers, provided you include silica gel packs to absorb any humidity. Check the silica and replace it if you notice discolouration or moisture.
- Exposing optics to short periods of sunlight or artificial UV light may help prevent fungal growth.
- Use replaceable fungicidal pellets in cabinets where optics are stored or inside large devices such as surgical microscopes. These can be obtained from some instrument manufacturers, and have a useful life of about 3 years.
- If it is difficult to keep the environment dry you can construct a drying box (see Figure 2) for storing the optical components of your equipment when they are not in use. The box consists of a heater or light bulb used to heat up and dry the air. Openings at the top and bottom permit air exchange in the box with air flowing from bottom to top. Mesh (dust screen) filters placed on the openings will prevent dust from entering the box.

## Removing fungus

Removing fungus from lenses can be difficult and may not yield suitable results since the damage is often permanent. Killing and removing the fungus and cleaning the optical surface may prolong the useful life of the instrument, however, if it can still provide an acceptable image.

### Required materials:

- Fungicide. Optical fungicide solutions tend to be expensive and hard to obtain, but they are available from some optical equipment manufacturers. Alternatively, you can use a 50/50 mix of hydrogen peroxide (H_2_O_2_) and ammonia (NH_3_). Usually, 5 ml of each is adequate (10 cc in total). Mix just prior to use and do not store the mixed product.
- Small (5 ml) syringe
- Lens cleaning solution
- Cotton-tipped swabs and lens tissue paper.

**Figure:**
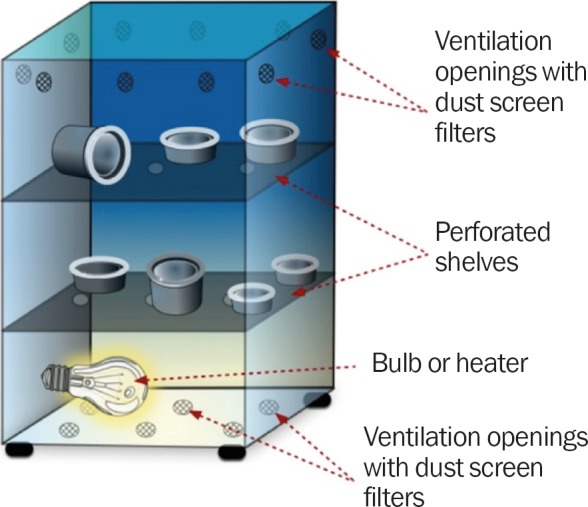
Figure 2

### Procedure

- If possible, remove the optical components requiring fungus removal and place them on a clean, flat work surface.
- Dip the cotton-tipped swab in the fungicide mix and set aside.
- Using a small (5 ml) syringe without a needle, place a few drops of fungicide on the affected optical surface. Use the cotton-tipped swab to spread the drops over the surface. Repeat until the entire optical surface is coated with a thin layer of fungicide.
- Let the fungicide do its work for about 1 hour. Check periodically and re-apply fungicide if the previous application has dried out.
- When the hour has passed, gently apply a dry lens tissue directly on the optical surface to absorb the fungicide. **Do not** rub the tissue on the optical surface. Remove the lens tissue and re-apply until there is none left.
- Allow to dry for 1 hour.
- Clean the optical surface two or three times using the standard method for cleaning optics.
- Re-examine the optical surface for evidence of active fungus. **Note:** You will see the evidence of damage caused by the fungus; this is permanent and not reversible.
- It is possible that fungus has attacked the internal components of a lens assembly. Do not disassemble any optical assemblies (such as multi element lenses and oculars or eye pieces), but refer repairs to the manufacturer's qualified service representative.
- Once all the optical surfaces requiring fungus removal have been treated, reassemble the equipment.
- As there is a high risk of recurrence, carefully examine the equipment on a regular basis, or at least once a month. Re-apply fungicide if you see evidence of recurrence.

